# Understanding Internalized Stigma’s Role in Sex-Specific Suicidal Ideation among Individuals with Bipolar Disorder

**DOI:** 10.3390/jcm13144000

**Published:** 2024-07-09

**Authors:** Martina D’Angelo, Luca Steardo

**Affiliations:** Psychiatry Unit, Department of Health Sciences, University of Catanzaro Magna Graecia, 88100 Catanzaro, Italy; steardo@unicz.it

**Keywords:** bipolar disorder, internalized stigma, suicide, sex differences

## Abstract

**Background**: The experience of stigma can exert a profound impact on the mental health and well-being of individuals with bipolar disorder (BD). Our study explores which factors of internalized stigma are associated with suicidal ideation and how they differ between the two sexes in a clinical sample of BD patients. **Methods**: The study follows a cross-sectional study design, employing the Clinical Global Impression for Bipolar Patients (CGI-BP) to evaluate the overall severity of illness and the alteration in patients affected by bipolar disorder, the Internalized Stigma of Mental Illness (ISMI) assessing self-stigma among individuals experiencing mental disorders, and the Columbia Suicide Severity Rating Scale (C-SSRS) identifying and assessing individuals vulnerable to suicide. Descriptive analyses, analysis of variance (ANOVA), and logistic regression analysis were conducted, and 344 BD subjects were recruited. **Results**: Our ANOVA results revealed a significant association between sex and suicide across ISMI sub-items (*p* = 0.000). Logistic regression analysis comprised three phases: Discrimination was consistently significant across all phases (*p* < 0.001), while Alienation and Stereotype emerged as additional predictors of suicide in later phases of the analysis (*p* < 0.001). **Conclusions**: Our study contributes to the growing body of literature on internalized stigma, sex, and suicidality among individuals with bipolar disorder. Early intervention programs and prevention strategies are needed.

## 1. Introduction

Internalized stigma in mental illness, in which individuals diagnosed with bipolar disorder (BD) or other mental health conditions internalize and accept societal prejudices and discrimination toward their illness, resulting in feelings of shame, guilt, or self-denigration [1,2]. This can have a significant impact on the mental health and psychological well-being of patients, leading to a range of negative outcomes, including suicidal ideation and behaviors [3,4,5]. Among women diagnosed with psychiatric disorders, it has garnered significant attention in recent years, both in research and clinical practice. The association between stigma and suicide in women with bipolar disorder is of great clinical and scientific significance, as both factors significantly contribute to the disease burden and the risk of adverse outcomes [6,7]. Mental health conditions, such as bipolar disorder, often carry a heavy burden of stigma and discrimination, both externally from societal attitudes and internally within individuals themselves [8,9]. This internalized stigma can profoundly impact individuals’ self-perception, overall quality of life, treatment adherence, and, most critically, their risk of suicide [10,11]. A growing body of literature has delved into the intricate interplay between sex, mental illness, and internalized stigma [12]. Studies have elucidated the distinct challenges confronted by women with psychiatric disorders as they navigate societal stereotypes, discrimination, and the weight of stigma associated with their condition [13,14]. Moreover, the intersectionality of sex with other social determinants, including socioeconomic status, ethnicity, and sexual orientation, further complicates the experience of stigma and its ramifications for women living with mental illness [15,16]. The experience of stigma can profoundly impact the mental health and well-being of women with bipolar disorder [14,17]. Stigma exacerbates symptoms of depression and anxiety, increases social isolation, and contributes to low self-esteem and hopelessness [18]. These psychological effects significantly elevate the risk of suicidal ideation and self-harming behaviors [19]. Studies have demonstrated that individuals perceiving higher levels of stigma are more prone to experiencing suicidal ideation and engaging in self-harming behaviors [13,20,21,22]. Previous research has suggested that women with bipolar disorder may experience and internalize stigma differently from men, potentially influencing their risk of suicide [17,23]. Women with bipolar disorder often face unique societal and cultural expectations regarding mental health, which can contribute to heightened stigma and increased internalization of negative beliefs about their condition [24]. This internalization process can exacerbate feelings of shame, self-blame, and social isolation, which are known risk factors for suicidal ideation and behavior [25]. Additionally, sex-specific roles and norms may influence how individuals perceive and respond to stigma, with women more likely to internalize negative stereotypes about mental illness due to societal pressures related to femininity and emotional expression [26]. These studies have underscored the pervasive nature of stigma across various facets of life, encompassing relationships, employment, healthcare, and social interactions [27]. Furthermore, research has shed light on the psychological mechanisms through which internalized stigma exacerbates adverse outcomes, including heightened symptom severity, diminished treatment-seeking behavior, and increased susceptibility to suicidal ideation and behavior [7,28,29]. Several studies have indicated that experiences of discrimination exacerbate suicidal ideation through self-stigmatization, social isolation, and feelings of despair [21,30]. Recent investigations have evaluated interventions aimed at mitigating internalized stigma and its detrimental effects on individuals with mental illness [31,32].

In this context, the present study contributes to the burgeoning literature by examining the association between internalized stigma, sex, and suicidality among individuals diagnosed with bipolar disorder. Through a comprehensive statistical analysis, we aim to elucidate the nuanced relationships between internalized stigma and all its variables in shaping suicidal ideation among women and men with bipolar disorder. For this reason, we hypothesize that the perception of internalized stigma may impact the development of suicidal ideation in patients with BD and that this may differ between the two sexes. Therefore, the present study aims to assess which factors of internalized stigma are associated with suicidal ideation and how they differ between the two sexes in a clinical sample of BD patients, conducting a cross-sectional study design.

## 2. Materials and Methods

This research was conducted as a naturalistic, uncontrolled, cross-sectional observational study at the outpatient Psychiatry unit of the University Hospital Mater Domini in Catanzaro (Italy) conducted from April 2020 to December 2023. All consecutive potential candidates were assessed for eligibility and invited to join the study. The enrolled patients were outpatients who received regular monitoring and maintained stable therapy over time. Participants underwent evaluation and diagnosis via a clinical interview administered by experienced clinicians who utilized the Structured Clinical Interview for DSM-5 (SCID-5-CV). The interviewers were experienced psychiatrists actively involved in clinical research and skilled in administering neuropsychiatric evaluations and regularly employed these tools daily. The inclusion criteria were as follows: (1) aged between 18 and 65 years, with the ability to read and understand the informed consent form; (2) capable of completing self-report questionnaires; (3) diagnosed with BD type-I (BD-I) or type-II (BD-II); and (4) clinically stable, as demonstrated by a Clinical Global Impression for Bipolar Patients (CGI-BP) score of ≤2 on item 1 (severity of illness) at enrollment. No additional inclusion criteria were set to capture a real-world clinical sample reflective of routine clinical activity. The exclusion criteria were as follows: (1) recent (≤6 months) or uncertain BD diagnosis or a medical history that was implausible or undocumented; (2) comorbid psychiatric diagnoses (e.g., schizophrenia spectrum disorders, post-traumatic stress disorder); (3) dementia or intellectual disability from mild to severe according to DSM-5 (IQ < 70); and (4) severe medical conditions associated with psychiatric symptoms (e.g., temporal lobe epilepsy, multiple sclerosis, brain trauma). In line with the Ethical Committee’s guidelines, participants received a comprehensive explanation of the study’s aims and methods and provided written informed consent before any procedures. The study protocol was submitted to and approved by the Ethical Committee of the University Hospital Mater Domini in Catanzaro (n.307/2020). The study was conducted in accordance with the ethical principles of the revised Helsinki Declaration.

### 2.1. Psychometrics Tools

We gathered patients’ demographic and clinical information (e.g., psychopharmacological treatment) using a specifically designed schedule. In detail, we employed a semi-structured interview to collect data on age, sex, marital status, years of education, current occupation, family history of psychiatric disorders, psychiatric and general medical comorbidities, onset and longitudinal course of the disorder (e.g., number of depressive/hypomanic/manic episodes, mixed and anxious features, and psychotic symptoms), number of previous suicide attempts, suicidal ideation at the time of recruitment, psychiatric hospitalizations, and current prescribed treatments. Subsequently, all participants were assessed using the following Italian versions of evaluation scales:

The Clinical Global Impression for Bipolar Patients (CGI-BP) is a revised version of the original CGI, specifically created to evaluate overall illness severity and changes in individuals with bipolar disorder. This tool has previously been used with Italian clinical populations [33]. It consists of two parts: severity of illness and global improvement. Both parts are scored on a scale from one (“normal, not at all ill”) to seven (“among the most extremely ill patients”), with a score of zero indicating the inability to evaluate. The CGI-BP provides a comprehensive assessment of the patient’s condition, enabling clinicians to monitor the disorder’s severity over time and assess the effectiveness of treatments [34].

The Internalized Stigma of Mental Illness (ISMI) scale is a validated self-report instrument developed to assess the internalization of stigmatizing beliefs among individuals with mental illness [35]. The scale comprises 29 items across five subscales: Alienation, Stereotype Endorsement, Perceived Discrimination, Social Withdrawal, and Stigma Resistance. Each item is rated on a 4-point Likert scale, with higher scores indicating greater internalized stigma. The ISMI is widely used in research to assess the psychological impact of stigma on individuals with mental health conditions. It is a commonly used and validated measure, with its psychometric properties thoroughly assessed across various versions, cultures, and languages, including Italian, as well as in various significant mental health conditions (e.g., depression, schizophrenia, substance abuse, eating disorders) and general medical conditions (e.g., epilepsy, inflammatory bowel disease, leprosy). It is important to note that the five stigma resistance subscale items are reverse-coded and function as a validity check. Consequently, stigma resistance demonstrates a correlation pattern consistent with the other four subscales. A high overall score on the ISMI scale suggests greater severity of internalized stigma [36].

The Columbia Suicide Severity Rating Scale (C-SSRS) is a structured tool designed to evaluate suicidal ideation and behavior systematically. It is extensively utilized in clinical and research settings to gauge the severity and intensity of suicidal thoughts and actions [37]. The scale assesses four dimensions: the seriousness and intensity of thoughts, actions, and the likelihood of death. It begins with initial questions that, if answered affirmatively, lead to follow-up questions for further detail. The C-SSRS includes ten categories, each requiring a binary response (yes/no) to indicate the presence or absence of specific behaviors. These categories range from a wish to be dead to completed suicide. The outcome is a numerical score derived from these categories. Interpretation can occur at various levels, including itemized assessment, categorical scale, or overall severity. Specific ratings such as the lethality of suicidal behavior, suicide ideation score, and suicidal ideation intensity rating can be derived. Ultimately, interpretation relies on a thorough clinical evaluation, patient history, and clinical expertise [38].

### 2.2. Statistical Analysis

Descriptive analyses were conducted to assess the distribution of variables across the entire sample. Results were presented as frequencies for categorical variables and as mean ± standard deviation (SD) or median. Analysis of variance (ANOVA) was employed to examine the association between the dependent variable “suicide” and sub-items of the ISMI scale among different groups based on sex. Additionally, logistic regression analysis was performed with suicide as the dependent variable and sex as a covariate. This analysis was conducted in three phases to explore the predictive capacity of various variables included in the model, namely “Discrimination”, “Alienation”, and “Stereotype”, on the likelihood of suicide.

## 3. Results

In this study, we conducted a thorough statistical analysis to investigate the factors associated with suicide among 344 individuals diagnosed with bipolar disorder type I and type II. Our analysis encompassed a broad range of demographic characteristics, clinical variables, and psychosocial factors. Examining the demographic and clinical characteristics of our participants (Table 1), we found that the average age was 46.88 years, with an onset age of 25.73 years. The duration of untreated illness averaged 5.84 years. Participants reported experiencing an average of 5.81 depressive episodes, 4.04 manic episodes, and 2.65 hypomanic episodes. We assessed various psychosocial factors using specific scales, including Alienation, Stereotype, Discrimination, Social distancing, and Stigma resistance. Mean scores for these factors were as follows: Alienation 13.88 (±3.83), Stereotype 14.83 (±4.07), Discrimination 12.26 (±4.34), Social distancing 13.28 (±3.4), and Stigma resistance 9.08 (±1.87).

The ANOVA analysis (Table 2) yielded statistically significant associations between predictor variables and suicide outcomes. Notably, a significant relationship emerged between sex and suicide across several ISMI sub-items, such as alienation (*p* = 0.000), discrimination (*p* = 0.000), and stigma resistance (*p* = 0.000). These results were corrected for age.

Furthermore, our regression analysis (Table 3) comprised three phases aimed at exploring the predictive power of various factors. In Phase 1, Discrimination emerged as a significant predictor of suicide (*p* = 0.000). In Phase 2, Alienation showed a positive association with suicide, while Discrimination remained a significant predictor (*p* < 0.001). Finally, in Phase 3, Discrimination emerged as a significant predictor of suicide (*p* < 0.001).

The binary logistic regression analysis represented in Table 4 reveals that the associations between the independent variables and suicide exhibit significant variations across the sexes. Specifically, for females, a statistically significant relationship is observed between stereotypes (*p* < 0.001) and discrimination (*p* < 0.001) with suicide. Conversely, for males, a significant association is found between alienation and suicide (*p* < 0.001). These findings suggest that specific psychosocial factors, such as stereotypes and discrimination for females and alienation for males, may significantly influence the likelihood of suicide, indicating notable sex-specific variations in risk factors.

### Dependent Variable: Suicide

Overall, our analysis underscores the importance of considering both clinical and psychosocial factors when assessing suicide risk among individuals with bipolar disorder. Factors such as discrimination, alienation, and stereotypes are pivotal and should be addressed in suicide prevention and intervention strategies tailored to this vulnerable population. The statistical results indicate alienation, discrimination, and stereotype are all significantly associated with suicidal ideation. Binary logistic regression analysis did not yield statistically significant differences between sexes.

## 4. Discussion

To the best of our knowledge, this is the first study that delved into the intricate relationship between internalized stigma, sex, and suicidal ideation among individuals diagnosed with bipolar disorder. Through a comprehensive statistical analysis, we explored the nuanced interplay between internalized stigma in shaping suicidal ideation highlighting the differences between males and females within this vulnerable population. Our investigation yielded significant findings that warrant careful consideration and interpretation in the context of existing literature and clinical practice. The main finding that emerges from this study is how suicidal ideation is closely associated with certain sub-items of internalized stigma: Alienation, Stereotype, and Discrimination in BD. Additionally, differences in the perception of stigma between the two sexes emerge, which subsequently may lead to the onset of suicidal ideation. Furthermore, our results, for the first time, demonstrate a difference in perceiving stigma between men and women. Statistical analysis reveals that women are more vulnerable to stereotypes and discrimination. These results are not to be underestimated because they can easily lead fragile individuals to suicidal ideation. Feeling discriminated against by peers and encountering the stereotypes often attributed to women can lead to anger, frustration, isolation, and consequently, the onset of suicidal ideation [30]. On the other hand, regarding the male sex, we have a stronger association with the sub-item of alienation. These data are important because, as we have already discussed in our previous scientific work [39], this item is often associated with dissociation in bipolar disorder, with all the consequences it brings, including a worse prognosis, the onset of psychotic symptoms, and suicidal ideation [40]. Our assessment of internalized stigma using specific scales provided valuable insights into the experiences of individuals living with bipolar disorder. The mean scores for Alienation, Stereotype, Discrimination, Social distancing, and Stigma resistance shed light on the pervasive nature of internalized stigma and its multifaceted impact on individuals’ lives. These findings align with previous research highlighting the profound influence of stigma on self-perception, social relationships, treatment adherence, and overall well-being among individuals with mental illness [41,42]. Women may face unique challenges associated with sex-based stigma and its intersection with other social determinants, including socioeconomic status, ethnicity, and sexual orientation [43].

The relevance of sex differences in this context is particularly significant due to the distinct societal and psychological pressures experienced by men and women, which have been extensively documented in scientific literature [44]. Women with bipolar disorder often face unique gender-specific stigma, such as being perceived as overly emotional or unstable [45]. This perception can exacerbate feelings of discrimination and alienation, impacting their self-esteem and social interactions [30]. The results of the study indicate a clear trend toward a higher prevalence of women compared to men in every sample and subsample of BD I and BD II analyzed. This supports the hypothesis that BD diagnoses are increasing more significantly in women than in men [45]. Women with BD often exhibit specific clinical characteristics that distinguish them from men, including higher rates of rapid cycling, more frequent depressive polarity, and a greater number of suicide attempts [46,47]. These factors may contribute to misdiagnoses of MDD instead of BD, leading to delays in appropriate treatment. According to studies, such stereotypes not only affect women’s self-perception but also their willingness to seek help, thereby increasing their risk of suicidal ideation [48]. Research has shown that women are more likely to internalize stigma, which can lead to increased depressive symptoms and higher rates of suicidal thoughts and behaviors [49]. Men, on the other hand, may struggle with societal expectations to appear strong and self-reliant, which can lead to a greater sense of isolation and alienation when they experience mental health issues [50]. This is supported by research indicating that men are less likely to seek help for mental health problems due to fears of appearing weak or vulnerable [51]. Such societal pressures can result in men experiencing higher levels of internalized stigma related to feelings of inadequacy and failure, which are closely linked to suicidal ideation [52]. For instance, interventions for women might focus on enhancing self-esteem and empowerment, challenging gender-based stereotypes, and providing support for comorbid conditions. For men, interventions could emphasize the importance of seeking help, reducing the stigma associated with vulnerability, and providing alternative coping strategies that do not rely on substance use [51]. Recognizing and addressing these differences can improve the effectiveness of stigma reduction strategies and ultimately contribute to better mental health outcomes for all individuals with BD [53].

These findings underscore the importance of adopting a comprehensive approach to suicide prevention that addresses the complex interplay of clinical and psychosocial factors contributing to suicidal ideation and behavior [54,55,56]. Our findings are consistent with previous research and may be indicative of poorer psychosocial outcomes and lower functioning, given that occupational status is listed among the domains in the brief assessment of functioning, in women suffering from bipolar disorder. Moreover, in our recent study, it was highlighted that women diagnosed with bipolar disorder showed a worse response to treatment with anticonvulsants [57]. Overall, these findings highlight the importance of considering gender differences in the assessment and management of bipolar disorder.

### Implications for Clinical Practice and Research

The findings of our study have important implications for clinical practice, research, and public health initiatives aimed at addressing internalized stigma and preventing suicide among individuals with bipolar disorder. From a clinical perspective, our results underscore the importance of incorporating a systematic assessment of internalized stigma and suicidal ideation into comprehensive psychiatric evaluations of individuals with bipolar disorder. Several studies have demonstrated that internalized stigma is associated with increased symptom severity and decreased treatment adherence among individuals with bipolar disorder [1,58,59,60]. Therefore, early identification of this condition can be crucial for improving clinical outcomes. Furthermore, our research highlights the need for personalized interventions aimed at reducing internalized stigma, fostering social connection, and enhancing coping skills among individuals with bipolar disorder. Various intervention modalities have shown promise in this regard. For example, cognitive-behavioral therapy (CBT) can assist individuals in identifying and modifying distorted thoughts related to stigma [61,62,63]. Peer support programs can provide a network of emotional and practical support [64]. Community-based initiatives can help reduce social isolation and promote greater engagement in daily life [65]. Moreover, our study highlights the need for tailored interventions aimed at mitigating internalized stigma, fostering social connectedness, and enhancing coping skills among individuals living with bipolar disorder. In addition, our findings underscore the importance of longitudinal studies to elucidate the trajectories of internalized stigma and suicidal ideation over time and their implications for treatment outcomes and recovery [66]. Longitudinal research can provide valuable insights into the dynamic nature of internalized stigma and inform the development of targeted interventions to address stigma-related barriers to recovery among individuals with bipolar disorder [10,67,68,69]. Furthermore, research focusing on culturally sensitive approaches to stigma reduction and resilience-building strategies among diverse populations of women with bipolar disorder is warranted [70]. Intersectional approaches that acknowledge the unique experiences and needs of women from diverse cultural backgrounds can enhance the effectiveness and relevance of stigma reduction interventions and suicide prevention strategies [71]. The results presented in our study should be interpreted in the light of some limitations. First, this study involved a cross-section of a large proportion of the psychiatric population, which prevents establishing definite causal relationships. Secondly, the utilization of a cross-sectional design employing self-administered evaluations entails a structural constraint concerning the compilation and dependability of the data, necessitating careful consideration in any extrapolation of the findings. An additional limitation of our study is the use of self-reported scales, which introduces a potential recall bias in the cohort of patients recruited. The obtained results could serve as a robust foundation for prospective longitudinal investigations, which could offer a more comprehensive understanding of the longitudinal dynamics of stigma-related barriers among individuals with bipolar disorder. These studies could also inform the development of evidence-based, targeted interventions aimed at effectively addressing such barriers, thereby contributing to the promotion of recovery and enhancement of the quality of life for this population.

## 5. Conclusions

In conclusion, our study contributes to the growing body of literature on internalized stigma, sex, and suicidality among individuals with bipolar disorder. By elucidating the complex interplay of demographic characteristics, clinical variables, and psychosocial factors in shaping suicidal ideation, our findings provide valuable insights for clinicians, researchers, and policymakers working to address the mental health needs of individuals living with bipolar disorder. This study’s limitations include the inability to establish causal relationships due to its cross-sectional nature within a large psychiatric population. Additionally, the use of self-administered assessments introduces potential biases, particularly recall bias among the patient cohort. Through targeted interventions and suicide prevention strategies tailored to the needs of individuals grappling with bipolar disorder and experiencing internalized stigma, we can enhance mental health equity, reduce stigma, and improve outcomes for individuals living with bipolar disorder worldwide. Moving forward, continued efforts to address internalized stigma and promote resilience among individuals with bipolar disorder are essential for advancing mental health equity and enhancing the well-being of individuals and communities affected by bipolar disorder.

## Figures and Tables

**Table 1:** Socio-demographic characteristics of the sample (*n* = 344 patients).

Characteristic	M or *n*	±SD or %
Age, M (SD±)	46.88	±13.94
Age at onset, M (SD±)	25.73	±9.40
Duration of untreated illness, M (SD±)	5.84	±10.31
Number of depressive episodes, M (SD±)	5.81	±5.92
Number of manic episodes, M (SD±)	4.04	±4.41
Number of hypomanic episodes, M (SD±)	2.65	±2.69
Numbers of total affective episodes, M (SD±)	11.39	±9.87
Alienation, M (SD±)	13.88	±3.83
Stereotype, M (SD±)	14.83	±4.07
Discrimination, M (SD±)	12.26	±4.34
Social distancing, M (SD±)	13.28	±3.4
Stigma resistance, M (SD±)	9.08	±1.87
Female, N (yes%)	177	51.5%
Graduation, N (yes%)	246	71.5%
Marital status, N (yes%)	186	54.1%
Employed, N (yes%)	171	49.7%
Diagnosis of bipolar disorder I, N (yes%)	253	73.5%
Diagnosis of bipolar disorder II, N (yes%)	91	26.5%
Family history of psychiatric disorder, N (yes%)	190	55.2%
Seasonality, N (yes%)	171	49.7%
Aggressive behaviors, N (yes%)	194	56.4%
Mixed features, N (yes%)	193	56.1%
Lifetime abuse, N (yes%)	124	36.0%
Psychotic symptoms, N (yes%)	144	41.9%
Antidepressant mania, N (yes%)	85	24.7%
Suicide attempts, N (yes%)	138	40.1%

M: mean; *n*: total number; SD: standard deviation; %: percentage.

**Table 2:** Analysis of variance (ANOVA).

	Dependent Variable	Type III Sum of Squares	gl	Quadratic Mean	F	Sign.
Correct model	Suicide	46.782 ^a^	56	0.835	6.686	0.000
	Sex	56.936 ^b^	56	1.017	10.065	0.000
Intercept	Suicide	4.237	1	4.237	33.914	0.000
	Sex	1.829	1	1.829	18.105	0.000
Alienation	Suicide	9.319	1	9.319	74.590	0.000
	Sex	2.183	1	2.183	21.607	0.000
Stereotype	Suicide	0.962	1	0.962	7.702	0.006
	Sex	0.275	1	0.275	2.723	0.100
Discrimination	Suicide	2.152	1	2.152	17.224	0.000
	Sex	11.768	1	11.768	116.496	0.000
Social distancing	Suicide	1.813	1	1.813	14.511	0.000
	Sex	0.861	1	0.861	8.519	0.004
Stigma Resistance	Suicide	0.525	1	0.525	4.205	0.041
	Sex	2.032	1	2.032	20.114	0.000
Age	Suicide	14.366	51	0.282	2.255	0.000
	Sex	20.849	51	0.409	4.047	0.000
Error	Suicide	35.858	287	0.125		
	Sex	28.992	287	0.101		
Total	Suicide	138.000	344			
	Sex	167.000	344			
Correct Total	Suicide	82.640	343			
	Sex	85.927	343			
**Model Summary**						
**Phase**	**Log-Likelihood-2**	**Cox and Snell R-squared**		**Nagelkerke R-squared**		
1	49.595 ^c^	0.641		0.855		
2	44.055 ^c^	0.655		0.874		
3	36.180 ^d^	0.674		0.900		

^a^ R-squared = 0.566 (Adjusted R-squared = 0.481); ^b^ R-squared = 0.663 (Adjusted R-squared = 0.597). ^c^ Estimation terminated at iteration number 7 because parameter estimates were changed by less than 0.001. ^d^ Estimation terminated at iteration number 9 because parameter estimates were changed by less than 0.001.

**Table 3:** Regression analysis: results for suicide risk factors across phases.

		B	S.E.	Wald	Sign.	Exp(B)	95% C.I. Per Exp(B)	
							Lower	Upper
Phase 1 ^a^	Discrimination	−0.832	0.135	37.835	0.000	0.435	0.334	0.567
	Constant	11.966	1.974	36.748	0.000	157,337.529		
Phase 2 ^b^	Alienation	0.331	0.154	4.603	0.032	1.392	1.029	1.882
	Discrimination	−0.858	0.152	31.864	0.001	0.424	0.315	0.571
	Constant	6.785	2.730	6.178	0.013	884.373		
Phase 3 ^c^	Alienation	0.734	0.294	6.207	0.013	2.082	1.169	3.709
	Stereotype	−0.635	0	4.684	0.030	0.530	0.298	0.942
	Discrimination	−0.971	0.215	20.367	0.001	0.379	0.249	0.577
	Constant	13.533	4.462	9.197	0.002	754,135.825		

^a^ Variables entered in Phase 1: Discrimination. ^b^ Variables entered in Phase 2: Alienation. ^c^ Variables entered in Phase 3: Stereotype.

**Table 4:** Binary logistic regression analysis.

		B	S.E.	Wald	Sign.	Exp(B)	95% C.I. Per Exp(B)	
							Lower	Upper
Female	Stereotype	0.354	0.099	12.919	0.001	1.425	1.175	1.729
Female	Discrimination	0.749	0.134	31.017	0.001	2.114	1.624	2.751
Male	Alienation	0.607	0.098	38.580	0.001	1.834	1.515	2.222

## Data Availability

No datasets were generated or analyzed during the current study.
